# Supplementary material for: Patient-Led, Technology-Assisted Malnutrition Risk Screening in Hospital: A Feasibility Study
Source: Nutrients. 2024 Apr 12;16(8):1139. doi: 10.3390/nu16081139 (PMC11055004; doi:10.3390/nu16081139)
Supplement: Supplementary file 1 [file nutrients-16-01139-s001.zip › Supplementary File S1 - Patient satisfaction survey.pdf]

Supplementary File S1: Patient satisfaction survey

Instructions: These questions are about your experience with completing the nutrition screening survey on your bedside TV. Please read each question carefully and circle the answer that best suits. When finished, please check you've answered all questions.

Q1. What was your overall experience with completing the nutrition screen?

Very negative	Negative	Neutral	Positive	Very positive

Q2. How easy or difficult were the instructions to follow (on the screen)?

Very difficult	Difficult	Neutral	Easy	Very easy

Q3. How satisfied are you with the explanations and/or assistance provided by research personnel in completing this task?

Very dissatisfied	Dissatisfied	Neutral	Satisfied	Very satisfied

Q4. How burdensome was it to complete this task?

Very burdensome	Somewhat burdensome	Neutral	Not much burden	No burden at all

Comments (optional response) ____

____

____

____

____
