# Supplementary material for: Patient-Led, Technology-Assisted Malnutrition Risk Screening in Hospital: A Feasibility Study
Source: Nutrients. 2024 Apr 12;16(8):1139. doi: 10.3390/nu16081139 (PMC11055004; doi:10.3390/nu16081139)
Supplement: Supplementary file 1 [file nutrients-16-01139-s001.zip › Supplementary File S2 - patient interview guide.pdf]

Supplementary File S2: Patient satisfaction survey Patient semi-structured interview guide

Preamble: “Thanks for taking the time to talk with me today. In this interview, I am going to ask you a few questions about your experiences with completing the MST via your bedside computer. There are no right or wrong answers; we just want your honest opinions and feedback. Before we start, do you have any questions about the interview?”

Domain	Example questions	Example prompts
Affective attitude / Experience	Overall, what did you think about the electronic MST?<br>How was the process for you, overall?<br>Were there any aspects you didn’t like?	How easy/difficult was it to navigate to from the home page?<br>What was the wording / instructions like? Were they easy or difficult to understand?<br>Was the layout / presentation suitable? Was it easy to see?
Intention	How confident were you in completing the electronic MST?<br>Would you complete this in the future (i.e. if you are admitted to hospital again) if asked to by your doctor, dietitian or nurse?<br>Do you see any barriers to asking patients (or their family members) to complete the MST while in hospital?	Was there anything that could have made you more confident / helped you to complete MST?<br>Why/why not?<br>Is there anything that could help overcome these barriers?
Coherence / Perceived effectiveness	Did you understand the purpose of the MST?<br>Do you think the information you provided in the MST will be useful for your health care team here in hospital?	Was there anything you would have liked more information on?<br>Why/why not?
Burden / Opportunity costs	How much effort or burden was it to complete the electronic MST?	Did participating in this study impact your day or cause additional stress?
Ethical consequences	What are your thoughts on having patients (or their family) completing the MST, which is usually done by nurses?	Do you see any problems with having patients complete tasks such as this while in hospital?
