# Supplementary figures and images for: Patient-Led, Technology-Assisted Malnutrition Risk Screening in Hospital: A Feasibility Study
Source: Nutrients. 2024 Apr 12;16(8):1139. doi: 10.3390/nu16081139 (PMC11055004; doi:10.3390/nu16081139)

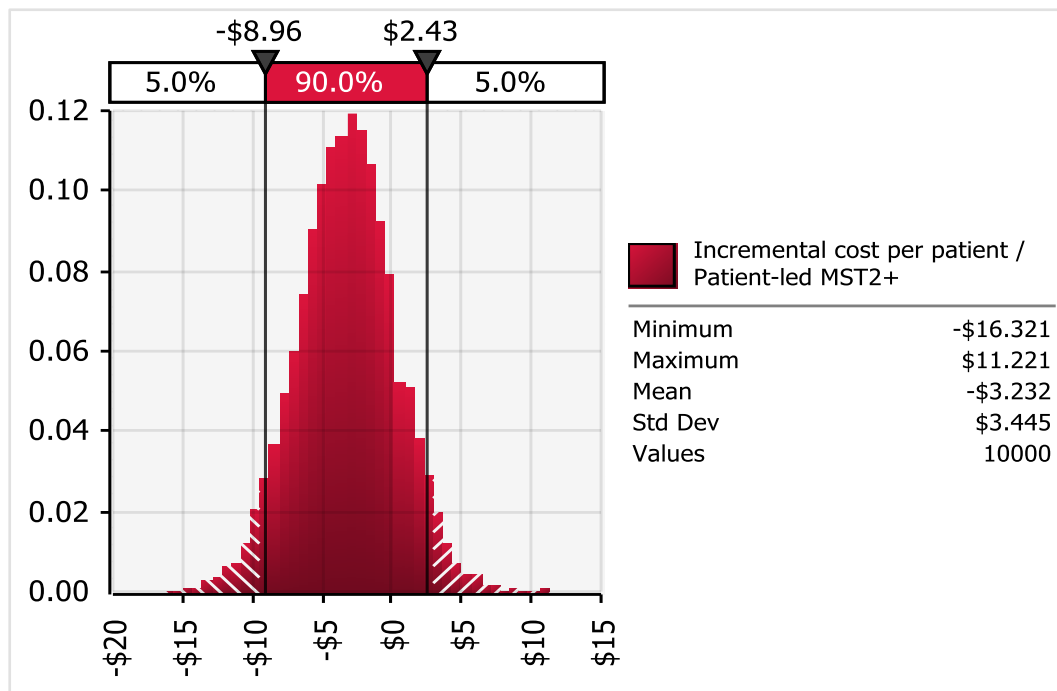

**Supplementary Figure S1:** Incremental cost per patient seen according to patient-led e-MST  
 $\geq 2$

Supplement: Supplementary file 1 [file nutrients-16-01139-s001.zip › Supplementary Figure S1.pdf]

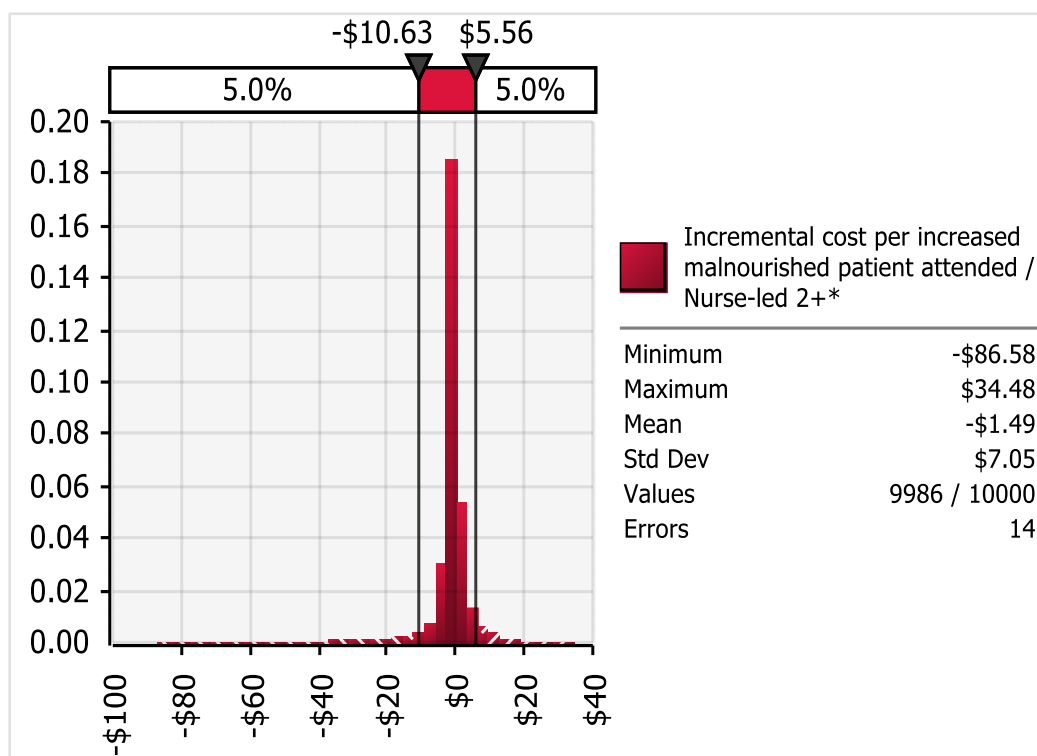

**Supplementary Figure S2:** Incremental cost per patient seen according to nurse-led MST  $\geq 2$

Supplement: Supplementary file 1 [file nutrients-16-01139-s001.zip › Supplementary Figure S2.pdf]
